# Value of cardiac computed tomography angiography in pre-operative assessment of infective endocarditis

**DOI:** 10.1186/s13019-019-0880-4

**Published:** 2019-03-12

**Authors:** Narumol Chaosuwannakit, Pattarapong Makarawate

**Affiliations:** 1 Radiology Department, Faculty of Medicine, Khon Kaen University, Khon Kaen Province, 40002 Thailand; 2 Cardiology Unit, Internal Medicine Department, Faculty of Medicine, Khon Kaen University, Khon Kaen Province, 40002 Thailand

**Keywords:** Infective endocarditis, Cardiac compute tomography, Valvular heart disease, Computed tomography angiography

## Abstract

**Background:**

Substantial development of cardiac computed tomography angiography (CTA) technology in the last decade has commanded to increase usage of this modality for assessing infective endocarditis (IE). The objective of this study is to evaluate the sensitivity and specificity of preoperative cardiac CTA imaging as opposed to transthoracic echocardiography (TTE) in the assessment of complications associated to IE, with comparison to surgical findings.

**Methods:**

Among 52 patients with surgically proven IE in our database, 24 underwent contrast-enhanced ECG cardiac CTA and were included in the study and all of them also underwent TTE.

**Results:**

For the detection of pseudoaneurysm/abscess in both native and prosthetic valves, cardiac CTA demonstrated significantly higher sensitivity (91.5% vs. 15.8%, *p* < 0.0001) with similar specificity (81.25). Cardiac CTA demonstrated similar sensitivity and specificity in identifying vegetation and valvular dehiscence in all patients.

**Conclusions:**

Preoperative cardiac CTA can be seen as complementary to TTE in assessing complications such as pseudoaneurysm or abscess of the patients with IE.

## Introduction

The diagnosis of infective endocarditis (IE) is challenging with up to 40% risk of mortality [1]. Early detection and accurate diagnosis is very significant for the care of patients with IE who will often require surgery along with antibiotic therapy. Transthoracic echocardiography (TTE) is typically the initial imaging study to assess valvular involvement in IE [2]. TTE offers good temporal and spatial resolution and is free of ionizing radiation. The important role of TTE in diagnosing IE has been valuable in the modified Duke criteria [3]. In contrast, cardiac computed tomography angiography (CTA), characteristically plays a supportive role in imaging assessment of complication of IE. Nonetheless, use of this modality for evaluating IE has been increasing particularly with the introduction of high resolution image acquisition [4–9, 11]. Recently, The European Society of Cardiology joint the use of cardiac CTA in its guideline for managing of IE [10]. At our institution, patients with planned cardiothoracic surgery often undergo a cardiac CTA to assistance in preoperative planning. Characteristically, we acquire preoperative cardiac CTA for the assessment of the complication of IE, proximity of cardiovascular structures to retrosternal regions and the existence and extent of coronary artery disease. Moreover, complications associated with IE may also be assessed on these CT images, with accusations for clinical management. In the present study, we anticipated to evaluate the role of cardiac CTA in the preoperative assessment of patients with recognized IE and to compare the results of cardiac CTA with those of TTE and intraoperative findings.

## Methods

Institutional Review Board approval was obtained for this retrospective review. Inclusion criteria were as follows; patients had surgically proven IE on gross examination and pathologically proven IE with positive causative organism on tissue culture and.

microscopic examination; and patients had cardiac CTA study for pre-surgical evaluation.

Exclusion criteria was the patients with negative culture and pathology findings from surgical tissue. All patients had TTE examinations. To assess true clinical performance of both.

CT and TTE imaging, we assessed only initial preoperative imaging in this investigation, to avoid bias. Imaging findings were analyzed separately based upon valve type (prosthetic and native) and anatomic position. We defined the following imaging criteria for IE complications.

### Echocardiography

Echocardiography was performed by expert cardiologist and key images were also recorded as video files. Conventional two-dimensional and Doppler echocardiography was performed using a commercially available ultrasound system with a 3–5 MHz, real-time, transthoracic echocardiographic transducer. Doppler B- and M-mode or 3D imaging was applied if clinically needed. Among all patients, 3D imaging was performed in 15 patients, including 3 patients with prosthetic valve infective endocarditis. The left ventricular functional parameters on. Echocardiography and the echocardiographic findings of infective endocarditis were evaluated.

### Cardiac CTA image acquisition

Preoperative cardiac CTA examinations were performed on a dual-source CT scanner (Definition FLASH, Siemens Healthcare, Forchheim, Germany). The system is equipped with two X-ray tubes and two corresponding detectors mounted on a single gantry with an angular offset of 90°. The coronary CTA used automatic tube current modulation in x, y, and z directions (Care Dose 4D, Siemens Healthcare). The coronary CTA scan parameters were as follows: two X-ray sources, detector collimation 32 × 0.6 mm with double sampling by rapid alteration of the focal spot in the longitudinal direction (Z-flying focal spot), rotation time 330 ms, tube voltage 120 kV. Image acquisition was performed during inspiratory breath-hold. To familiarise the patient with the protocol, breath-holding was practiced before the examination. The scan was followed by a test bolus injection to calculate the peak of contrast enhancement time. Then the final cardiac CTA was taken. A bolus of iodinated contrast material (350 mg/ml, Omnipaque; GE Healthcare) at a dose of 1.5 ml/kg with dual-head power injector followed by 10–20 ml of saline flush at a same rate to that of the contrast injection. Axial images were reconstructed with 0.75 mm slice thickness and 0.5 mm increment using a medium sharp convolution kernel (B26) and retrospective ECG gating. The reconstructions were performed in 5% steps over the entire R-R cycle using a single-segment algorithm that utilized quarter segments of projection data from both detectors. Patients were scanned in the supine position [12].

### Cardiac computed tomography angiography image analysis

Cardiac computed tomography angiography (CTA) image analysis was performed retrospectively by a radiologist with 10 years of experience in examining cardiovascular CT scans and blinded to the clinical data. First, all axial image data are evaluated using a 3D post processing workstation with Syngo software (Siemens Healthcare). Various image reformatting techniques including curved planar reconstruction, maximum intensity projection (MIP), minimum intensity projection, and volume-rendering technique (VRT) are used to obtain all the clinically relevant information. The morphology of infective endocarditis was classified into categories as follows [13]:

#### Pseudoaneurysm

**Fig. 1:**
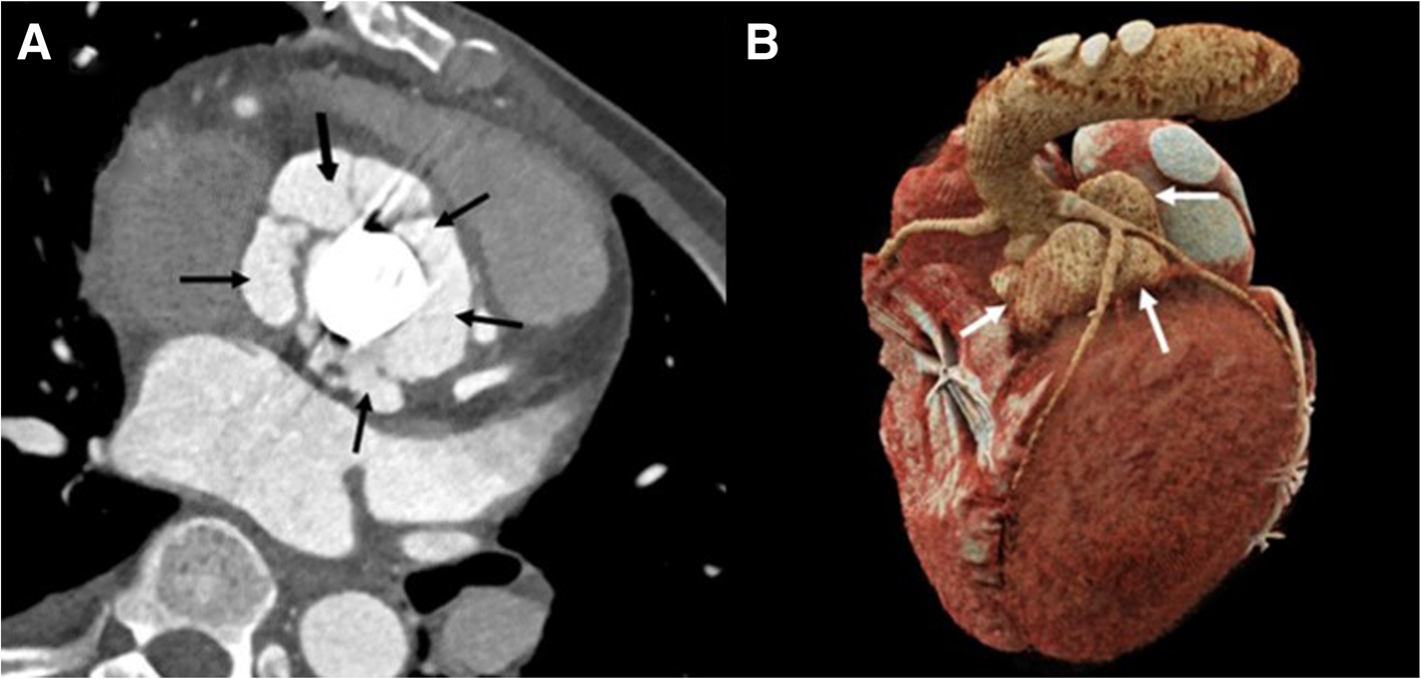
A 43-year-old man with a mechanical aortic valve. An aortic valve view (**a**) and cinematic reconstruction image (**b**) of cardiac CTA showing multiple paravalvular pseudoaneurysms (arrows). Noted the left anterior descending artery was located just above the pseudoaneurysm which demonstrated clearly by cardiac CTA. The pseudoaneurysm could not be detected on TTE due to the shadowing of the mechanical aortic valve

Contrast filled smooth walled sac adjacent to vascular structure or heart, usually with a visible direct connection (Fig. 1).

#### Abscess

Low attenuation central necrotic component within a peripheral organized rim, which may be irregular and thick, with enhancement by contrast. Surrounding inflammation.

and mass effect may also be present. Diffuse soft tissue thickening surrounding cardiac or aortic structures which may represent phlegmon/early abscess were also placed in this category.

#### Dehiscence

**Fig. 2:**
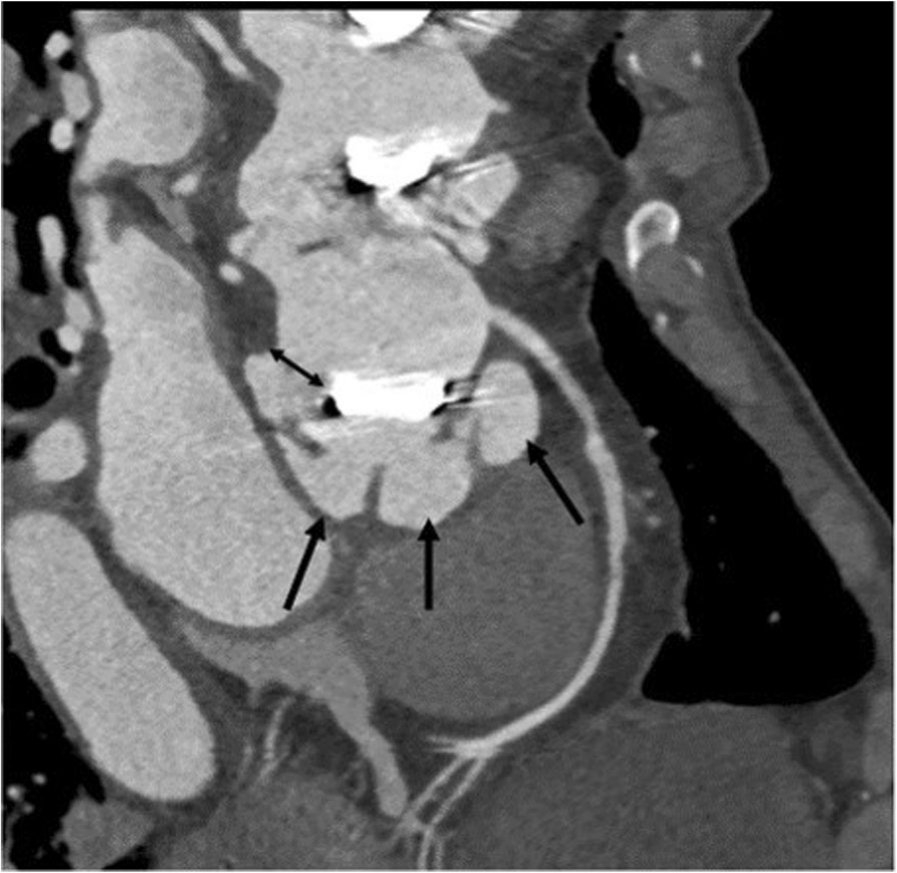
Dehiscence of a mechanical aortic valve in setting of endocarditis complicated by pseudoaneurysm (arrows) and malalignment of prosthesis with tissue defect between annulus and prosthesis representing dehiscence (double headed arrow)

Malalignment of prosthesis with tissue defect between annulus and prosthesis (Fig. 2).

#### Vegetation

**Fig. 3:**
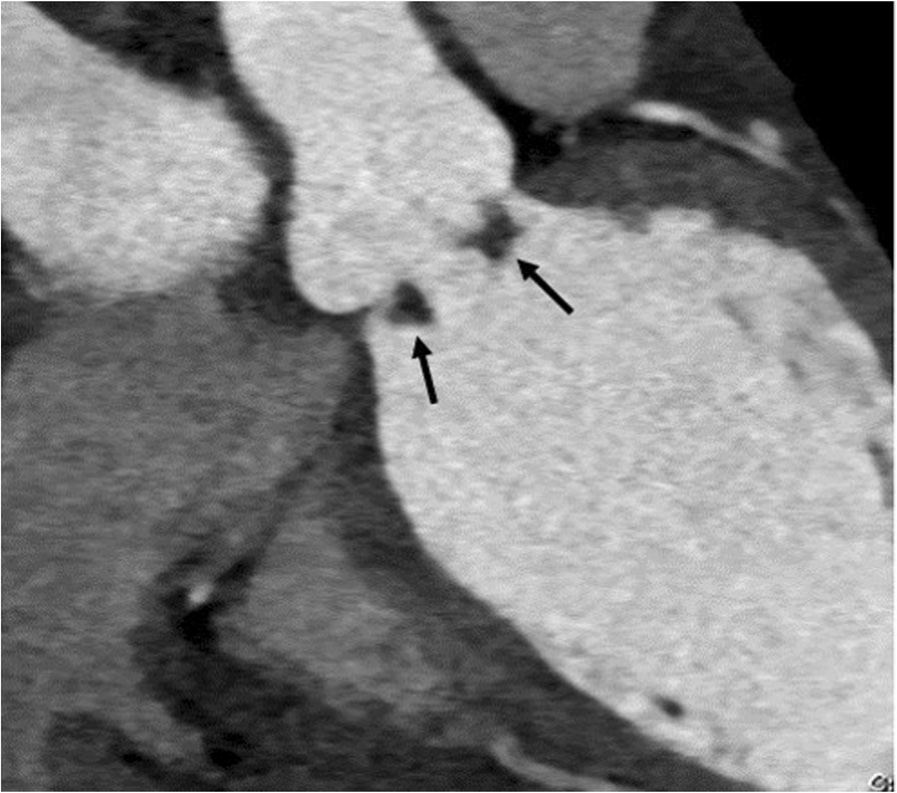
A 34-year-old man with rheumatic valve disease. On cardiac CTA showing nodular low-density lesions at aortic valve (arrow). Clinically, vegetation of rheumatic valve disease was suspected. In the operation, vegetation was noted in the rheumatic pathology of the aortic valve

Low to intermediate attenuation irregular mass or prominent focal thickening associated with valve, endocardium, or prosthesis (Fig. 3).

### Surgical findings

Cardiac surgeons reviewed the echocardiographic and CT findings before each operation, and the cardiac valves and paravalvular structures were closely evaluated during the operation by direct inspection and exploration. The location and extent of the involved site and the presence of vegetation, leaflet perforation, abscess/pseudoaneurysm, and/or paravalvular leakage were described in the operation records. Surgical treatments such as valve replacement or repair of infected sites and reconstruction with patches for abscess or paravalvular leakage were also recorded.

### Statistical analysis

Continuous data were expressed as mean ± SD. Statistical analyses were performed using SPSS software version 16 (SPSS, Inc., Chicago, IL, USA). A level of *p* < 0.05 was considered statistically significant and all reported *p*-values were two-sided. Sensitivity, specificity, negative predictive values (NPVs), and positive predictive values (PPVs) for pseudoaneurysm/ abscess, dehiscence, and vegetation were calculated using standard methodology for cases with definitive answers. McNemar’s test was used to evaluate for significant differences between modalities. A *P* value < 0.05 was indicative of statistical significance for all analyses.

## Results

A total of 24 patients with cardiac CTA scans performed during the patient’s hospitalization or within the previous week as part of preoperative assessment. The time period covers January 2015 to December 2017. The median interval between the cardiac CTA and TTE is 2 days. The median interval between cardiac CTA and surgery is 5 days. The median interval between TTE and surgery is 7 days. All study patients had surgical confirmation of IE involving the mitral and/or aortic valve, with causative organisms reviewed in each case. Cardiac CTA findings, including evidence of pseudoaneurysm, abscess, vegetation, and dehiscence were compared with TTE findings when relevant, with surgical reports used as the gold standard.

When comparing the performance of cardiac CTA and TTE, statistically significant differences were verified in the sensitivity for detection of pseudoaneurysm and abscess with cardiac CTA performing superiorly. The sensitivity and specificity for detection of vegetation and dehiscence did not differ significantly between the two groups. The findings remained unchanged when an analysis was limited to the subgroup of patients with prosthetic valve IE.(Table 1) Of the 5 patients who underwent intra-operative coronary artery bypass graft (CABG), none of them was evaluated with invasive cardiac catheterization pre-operatively.

**Table 1:** Evaluation of pseudoaneurysm, abscess, vegetation and dehiscence

Diagnostic performance	All			Native valve			Prosthetic valve		
	CT (*n* = 24)	TTE (n = 24)	*P*	CT (n = 14)	TTE (*n* = 14)	*P*	CT (n = 10)	TTE (*n* = 10)	*P*
Pseudoaneurysm/abscess (%)									
Sensitivity	91.5	15.79	< 0.0001	83.3	14.29	< 0.0001	100	16.67	< 0.0001
Specificity	81.25	81.25	1.0	87.5	85.7	0.43	75.0	75.0	1.0
PPV	84.5	50.0	0.0017	83.3	50.0	0.0023	85.7	50.0	0.0013
NPV	93.75	43.75	< 0.0001	87.5	50.0	0.0008	100	37.5	< 0.0001
Vegetation (%)									
Sensitivity	94.1	94.5	0.74	92.3	92.3	1.0	85.7	87.5	0.59
Specificity	66.67	50.0	0.49	50.0	66.67	0.49	66.67	33.3	0.0005
PPV	85.7	85.7	1.0	85.7	92.3	0.66	85.7	77.78	0.26
NPV	66.67	53.3	0.09	66.67	66.67	1.0	66.67	50	0.06
Dehiscence									
Sensitivity	66.67	66.67	1.0	66.67	66.67	1.0	66.67	66.67	1.0
Specificity	75.0	50.0	0.22	75.0	50.0	0.22	75.0	50.0	0.22
PPV	66.67	50.0	0.06	66.67	50.0	0.06	66.67	50.0	0.06
NPV	75.0	66.67	0.24	75.0	66.67	0.24	75.0	66.67	0.24

## Discussion

Cardiac CTA was similar to TTE in identification of vegetation and dehiscence in the present study, although, unsurprisingly, cardiac CTA demonstrated significantly higher sensitivity for pseudoaneurysm and abscess. This is regardless of almost one-half of patients having prosthetic valves with current evidence indicating that cardiac CTA has the capability to change diagnosis/treatment with echocardiography in 20–25% of prosthetic IE cases [14]. The diagnosis of perivalvular abscess/pseudoaneurysm related with endocarditis is vital because the presence of a perivalvular abscess/pseudoaneurysm increases the mortality rate by up to 2-fold [15]. In the present study, thirteen patients (54.2%) developed an abscess/pseudoaneurysm, and cardiac CTA correctly diagnosed ten more cases with abscesses/pseudoaneurysms than echocardiography. Echocardiography can underestimate perivalvular involvement, particularly in the case of small lesions, calcified or prosthetic valves [16, 17]. In patients with problematic to characterize perivalvular extension of infection by TTE, cardiac CTA is a practical next diagnostic step with the capability to define the coronary arterial anatomy concurrently, thus avoiding a supplementary procedure such as invasive coronary catheterization and contrast exposure. Cardiac CTA discovered paravalvular abscesses that might not be perceived on TTE due to shadowing of valvular calcification or mechanical valve. Cardiac CTA can be used as a subsidiary modality for detecting infective endocarditis, predominantly in patients who cannot tolerate transesophageal echocardiography (TEE) or with a poor acoustic window. Cardiac CTA has excellent negative predictive value in low to intermediate coronary artery disease (CAD) risk patients. The evidence has been less robust in high risk patients with some studies representing a continuous high negative predictive value whereas others sustaining a high sensitivity, but a decline in NPV due to the increased prevalence of disease [18, 19]. A cost effectiveness study integrating cardiac CTA as a first line test in pre-operative coronary evaluation for non-cardiac surgery established a decreased cost associated with the work-up and perioperative period [20]. Hence, along with avoiding an invasive procedure with a low risk for adverse events, this approach may be economically practical as well. This will reliably exclude significant coronary artery disease and possibly avoid invasive cardiac catheterization with its related potential complications, and might assistance with surgical planning. There are several studies published up to the present time to support this approach of pre-operative CTA in non-cardiac along with cardiac surgery. [21] Ciolina et al. demonstrated the additional value of cardiac CTA for assessing the aortic valve in pre-operative work-up for aortic stenosis [22]. In 42 patients, cardiac CTA appropriately graded aortic valve calcification, sized the aortic annulus and sinotubular junction, perceived thoracic aortic aneurysms, and properly assessed aortic valve area while also being used to evaluate coronary artery. The capability of cardiac CTA to concurrently provide additional diagnostic information and reliably evaluate coronary artery makes it a versatile diagnostic test with significant yield in the pre-operative work-up of patients with IE and other valvular disorders. Our study has limitations that warrant address. First, the analysis was performed in a single center for selected patients who undertook surgical treatment for infective endocarditis. A selection bias may consequently have been introduced during the evaluation of the results. Furthermore, because we have collected surgically managed patients in this study, we could not include all infective endocarditis patients who were treated by medical treatment alone. Nevertheless, we have focused on the surgical findings to compare the cardiac CTA and echocardiography findings. Second, surgical inspection might not be the faultless reference standard for infective endocarditis, predominantly for small lesions. Small vegetation, leaflet perforations, or abscess/pseudoaneurysm formation might not be noticed by surgical inspection.

Finally, the radiation dose is still the primary apprehension of cardiac CTA, particularly in retrospective ECG-gating with a higher radiation dose. A prospective ECG-triggering method using dual-source CT or iterative reconstruction might be used in a future study to reduce this radiation exposure.

## Conclusion

The sensitivities of cardiac CTA and TTE to perceive vegetation and dehiscence in patients with infective endocarditis are similar. Ten cases of abscess/pseudoaneurysm can be established by cardiac CTA alone. The greatest advantage of cardiac CTA in the setting of IE is its capability to couple the detection of complex cardiac anatomic abnormalities with coronary artery delineation. Cardiac CTA might be considered as an alternative coronary artery imaging modality in IE patients with low to intermediate risk of disease but meet guideline recommendations for coronary artery imaging.
